# Personalized exercise programs in oncology

**DOI:** 10.3389/or.2025.1645505

**Published:** 2025-09-12

**Authors:** Ahmed Yakdhan Saleh, Abdulkareem Shareef, Ashok Kumar Bishoyi, S. Renuka Jyothi, Rajashree Panigrahi, Amrita Pargaien, Gunjan Garg, Mashkhura Hafizova, Hayder Naji Sameer, Ahmed Yaseen, Zainab H. Athab, Mohaned Adil

**Affiliations:** ^1^ Department of Physical Education & Sport Sciences, College of Education Alnoor University, Nineveh, Iraq; ^2^ Ahl al bayt University, Kerbala, Iraq; ^3^ Department of Microbiology, Faculty of Science, Marwadi University Research Center, Marwadi University, Rajkot, Gujarat, India; ^4^ Department of Biotechnology and Genetics, School of Sciences, JAIN (Deemed to be University), Bangalore, Karnataka, India; ^5^ Department of Microbiology, IMS and SUM Hospital, Siksha ‘O’ Anusandhan (Deemed to be University), Bhubaneswar, Odisha, India; ^6^ Department of Pharmacy, Graphic Era Hill University, Bhimtal, India; ^7^ Centre for Promotion of Research, Graphic Era (Deemed to be University), Dehradun, Uttarakhand, India; ^8^ Centre for Research Impact & Outcome, Chitkara University Institute of Engineering and Technology, Chitkara University, Rajpura, Punjab, India; ^9^ Department of Pedagogy and Psychology, Samarkand State Medical University, Samarkand, Uzbekistan; ^10^ Collage of Pharmacy, National University of Science and Technology, Dhi Qar, Iraq; ^11^ Gilgamesh Ahliya University, Baghdad, Iraq; ^12^ Department of Pharmacy, Al-Zahrawi University College, Karbala, Iraq; ^13^ Pharmacy College, Al-Farahidi University, Baghdad, Iraq

**Keywords:** exercise, cancer, oncology, personalized, individualized, physical activity, survivorship, rehabilitation

## Abstract

Exercise is increasingly recognized as a safe and effective adjunct therapy across the cancer care continuum, offering improvements in physiological function, psychological wellbeing, and treatment outcomes. However, conventional one-size-fits-all exercise prescriptions often fall short of addressing the diverse needs of cancer patients, who differ significantly in tumor type, treatment modality, baseline fitness, and comorbidities. Personalized exercise programs offer a tailored, evidence-informed approach that enhances safety, adherence, and clinical benefits. This narrative review synthesizes the current literature on the physiological, psychological, and oncological impacts of exercise in cancer care, emphasizing the rationale, methodologies, and emerging tools for individualized exercise prescriptions. Integration of such programs into oncology practice requires standardized assessments, interdisciplinary collaboration, and digital infrastructure, with a focus on addressing barriers to implementation and ensuring equitable access. Personalized exercise programs have the potential to improve patient outcomes and survivorship experiences across diverse cancer populations.

## 1 Introduction

Globally, the burden of cancer continues to rise, with the latest GLOBOCAN estimates reporting approximately 20 million new cancer diagnoses and nearly 10 million cancer-related deaths in 2022 (1–4). Age-standardized incidence rates have climbed steadily over the past decade, driven by an aging population and lifestyle risk factors__, while mortality, although declining by about 1% per year since 2016, still contributes to high global healthcare and socioeconomic costs (5–7).

Evidence-based oncological treatments—surgery, chemotherapy, and radiotherapy—remain fundamental for tumor control but are accompanied by substantial short- and long-term toxicities, including profound fatigue, immunosuppression, musculoskeletal deconditioning, and neuropathies, which can significantly impair patients’ functional capacity and quality of life (8–11).

In response to treatment-related adverse effects, lifestyle interventions have gained prominence, with exercise emerging as a critical supportive strategy; randomized trials have demonstrated that structured exercise during chemotherapy and radiotherapy can enhance cardiorespiratory fitness, mitigate fatigue, and preserve lean body mass, reflecting a paradigm shift toward holistic cancer care (12–16). Extensive meta-analyses and systematic reviews have shown that aerobic and resistance training significantly reduce cancer-related fatigue, with a systematic review of 46 meta-analyses confirming a consistent benefit of exercise on fatigue reduction (17–21). In addition, exercise interventions have been associated with marked improvements in health-related quality of life metrics such as physical functioning and psychological wellbeing in multiple randomized controlled trials (22–24). A recent meta-analysis reported that higher muscle strength and cardiorespiratory fitness levels correlate with a 31%–46% lower risk of all-cause mortality in cancer patients, particularly in advanced-stage and lung and digestive cancers, underscoring potential survival benefits (25, 26).

Despite this robust evidence, traditional “one-size-fits-all” exercise prescriptions fall short of addressing the heterogeneous needs of cancer patients, who differ widely by tumor type, treatment regimen, baseline fitness, comorbidities, and personal preferences, leading to varying adherence and outcomes (8, 27, 28). Some research has highlighted that fixed exercise protocols may not optimize safety or efficacy across these diverse patient subgroups, signaling the need for more tailored approaches (29, 30).

Personalized exercise programs, in contrast, leverage individual assessments to customize the type, intensity, frequency, and duration of exercise, adapting prescriptions based on factors such as cardiopulmonary function, muscle strength, fatigue levels, and treatment phase (31, 32); a prospective study in lung cancer patients undergoing chemotherapy demonstrated that individualized exercise prescriptions led to significant improvements in 6-min walk distance, VO_2_ peak, muscle mass, and quality of life without adverse events (33). Conceptual frameworks and app-based platforms now exist to operationalize this personalization, enabling dynamic adjustment of exercise parameters in real-time to match patients’ evolving capabilities and treatment schedules (9, 34, 35).

The integration of exercise into cancer care has demonstrated significant benefits, including reduced fatigue, improved quality of life, and enhanced survival rates among cancer patients. However, traditional exercise prescriptions often adopt a one-size-fits-all approach, failing to account for the diverse needs and conditions of individual patients, particularly those in low-resource settings, rural communities, and marginalized populations. Disparities in access to exercise oncology programs—driven by socioeconomic status, geographic isolation, cultural barriers, and systemic inequities—threaten to widen existing gaps in cancer outcomes. Addressing these disparities is not merely an ethical imperative but a clinical necessity to ensure equitable translation of evidence into global practice (36). Recent studies highlight the importance of personalized exercise programs tailored to individual characteristics, such as cancer type, treatment phase, physical fitness, and comorbidities, to optimize outcomes and minimize risks (37).

While prior reviews have broadly examined the benefits of physical activity in oncology, few have focused specifically on how exercise prescriptions can be tailored to individual patient characteristics across the cancer care continuum. This narrative review addresses that gap by synthesizing current evidence on personalized exercise programming—defined as individualized prescriptions adjusted for tumor type, treatment phase, baseline physical function, comorbidities, and psychosocial factors. In doing so, this review bridges exercise physiology, implementation science, and survivorship care, providing a practical and conceptual framework to guide future clinical integration of personalized exercise in oncology.

## 2 Methodology

This narrative review was conducted using a structured approach to identify, select, and synthesize peer-reviewed literature on personalized exercise in oncology. We searched PubMed, Scopus, Web of Science, and Google Scholar using combinations of keywords such as “exercise,” “cancer,” “oncology,” “personalized,” “individualized,” “physical activity,” “survivorship,” and “rehabilitation.” We included English-language articles published between 2000 and 2025 that reported on physiological, psychological, or clinical outcomes of exercise interventions in cancer patients or survivors. Both clinical studies and implementation frameworks were considered. Exclusion criteria included animal studies without translational relevance, narrative pieces lacking primary data, and non-peer-reviewed sources. Reference lists of key reviews were manually searched for additional relevant studies.

## 3 Benefits of exercise in oncology

### 3.1 Physiological benefits

The role of physical activity in oncology has increasingly gained recognition as a safe and effective approach to enhancing physiological outcomes across the cancer care continuum (38, 39). Systematic reviews and meta-analyses highlight that exercise confers physiological advantages before, during, and after cancer treatment (39, 40).

In cancer prevention, engaging in regular physical activity is associated with a reduced risk of various cancers, attributed to beneficial physiological changes such as improved weight management, decreased systemic inflammation, enhanced insulin sensitivity, and regulation of sex hormones like estrogen (41, 42). While current evidence strongly supports aerobic exercise in this regard, data on the physiological benefits of resistance training for cancer prevention remain limited (42).

Exercise prior to treatment has also shown to yield positive physiological effects. A meta-analysis of 10 randomized controlled trials involving patients with non-small cell lung cancer reported that preoperative exercise improved functional capacity, alleviated dyspnea, shortened hospital stays, and decreased pulmonary complications (43, 44). These outcomes underscore the value of exercise in optimizing respiratory and overall physical function before medical interventions.

During cancer treatment, physical activity helps preserve physiological capacity by mitigating muscle wasting (sarcopenia) and improving cardiorespiratory fitness (45, 46). Animal studies further suggest that exercise may enhance the effectiveness of therapies like chemotherapy and radiotherapy by improving tumor oxygenation and vascular normalization, thereby supporting better physiological conditions in tissues (47). Although clinical confirmation is pending, these mechanisms provide a strong rationale for continued investigation (46).

In the context of immunotherapy, exercise is believed to modulate cellular immunity through the release of bioactive molecules such as interleukin-6 and epinephrine, which activate natural killer cells and T lymphocytes—contributing to a stronger physiological anti-tumor response (48, 49). Animal research also shows that physical activity may boost the efficacy of immune checkpoint inhibitors like PD-1/PD-L1 (50).

Post-treatment, exercise continues to provide physiological advantages. For example, a meta-analysis of 34 RCTs in breast cancer patients found significant reductions in insulin-like growth factor 1 (IGF-1), enhancements in muscle strength (e.g., bench and leg press performance), and decreases in BMI and body weight (51). Across various cancer types, benefits include improved aerobic capacity (VO_2_ max), muscle power, 6-minute walk distance, and handgrip strength, underscoring the sustained impact of exercise on physiological health after treatment (45).

Despite variability in participant characteristics (age, cancer type) and exercise regimens (intensity, duration), the positive influence of physical activity on physiological indicators remains robust (38, 39). However, short intervention durations (median 13 weeks) and insufficient detail on exercise intensity are common limitations (39).

In conclusion, substantial evidence supports the favorable physiological impact of exercise throughout all stages of cancer care. Notably, combining aerobic and resistance training often leads to greater improvements than aerobic activity alone (38–40).

### 3.2 Psychological and quality-of-life improvements

Exercise has also emerged as a key intervention for improving psychological wellbeing and quality of life (QoL) among cancer patients and survivors. Although variability exists in exercise modalities and participant populations, systematic reviews and meta-analyses consistently report positive effects (52–54).

In a systematic review of 40 RCTs involving 3,694 participants with diverse cancer types, Roland and Rogers (2018) found that interventions including resistance training, yoga, walking, Tai Chi, and Qigong significantly enhanced health-related QoL and reduced fatigue, anxiety, depression, and pain. However, changes in cognitive function and general health perception were not statistically significant. Despite some risk of bias due to limited blinding, the clinical relevance of these findings is evident (54).

Carayol et al. (2019) focused on women with breast cancer undergoing adjuvant therapy and reported improvements in fatigue, depression, and QoL, with borderline effects on anxiety. Interestingly, they identified a dose-response pattern in which moderate exercise (<12 MET-hours/week) had more pronounced benefits, indicating that lower-intensity programs may be particularly effective in this population (52).

McNeely et al. (2006) echoed these findings in their review of 14 RCTs involving breast cancer patients. Participants experienced improved QoL (measured via FACT-G and FACT-B), enhanced physical function, increased VO_2_ peak, and reduced fatigue. Notably, these gains were not accompanied by significant changes in BMI or body weight, suggesting that mental and functional improvements can occur independently of body composition shifts (53).

Community-based exercise programs also showed promising results. Knobf et al. (2014) conducted a supervised aerobic program for breast cancer survivors, demonstrating notable gains in physical, emotional, and social functioning, as well as reductions in fatigue, depression, and muscle stiffness over 4–6 months (55).

A comprehensive review by Battaglini et al. (2014), covering 25 years of literature, confirmed the wide-ranging benefits of both aerobic and resistance exercise in breast cancer survivors, including better cardiorespiratory fitness, reduced body fat, increased lean mass and muscular strength, and improvements in fatigue, depression, and QoL. This evidence supports the design of individualized exercise prescriptions based on treatment stage and patient needs (56).

Collectively, these studies confirm that exercise is a safe, effective, and accessible tool to enhance psychological health and QoL among cancer patients and survivors, despite heterogeneity in exercise type and dosage.

### 3.3 Treatment-related outcomes

Recent investigations have increasingly explored the impact of exercise on treatment-related outcomes in oncology. Both preclinical and clinical studies suggest that exercise can enhance the effectiveness of cancer therapies and contribute to improved patient wellbeing.

In preclinical settings, physical activity has been shown to augment the effects of chemotherapy and agents like tamoxifen across several cancer models. For instance, aerobic and resistance training improved cardiorespiratory fitness and lean body mass in breast cancer models, while enhancing chemotherapy sensitivity in melanoma and producing synergistic tumor volume reduction in Ewing sarcoma when combined with treatment (41).

Clinical data support these findings. In breast cancer patients receiving adjuvant chemotherapy, exercise has been linked to greater muscular strength, increased self-esteem, and improved treatment completion rates (57). Similarly, aerobic training has been associated with improved clinical responses in lymphoma patients (58). and in those with rectal cancer, exercise during neoadjuvant chemo radiation has been correlated with higher rates of complete or near-complete pathological response (59, 60).

Longitudinal evidence indicates that exercise may lower the risk of recurrence and enhance survival outcomes. Among breast cancer patients on adjuvant chemotherapy, physical activity has been associated with decreased recurrence and better disease-free survival (61). Additionally, resistance training has demonstrated potential to extend bone-related survival in patients with bone metastases (62).

Nevertheless, challenges persist. Many clinical trials suffer from small sample sizes and were not specifically designed to assess therapeutic efficacy (63). Differences in exercise protocols (type, intensity, duration) further complicate cross-study comparisons (57). Moreover, preclinical evidence suggests that the effect of exercise may vary based on tumor type and treatment; in one breast cancer model, for example, exercise diminished the efficacy of doxorubicin (63).

Overall, exercise appears to offer meaningful benefits in supporting cancer treatment outcomes. Still, more rigorously designed studies with larger cohorts are necessary to elucidate underlying mechanisms and refine exercise guidelines. Future research should also assess exercise’s role alongside therapies like immunotherapy and radiotherapy (61, 63).

## 4 Rationale for personalization

Personalizing exercise prescriptions in oncology is essential to address the marked heterogeneity among patients in terms of tumor biology, treatment exposures, baseline fitness, and comorbid conditions (27, 64). The following sections synthesize key findings from six seminal studies to elucidate why a one-size-fits-all approach is inadequate and to highlight how variability in clinical and patient factors must guide tailored exercise interventions.

### 4.1 Variability in cancer types and stages

Santa Mina and colleagues (2012) emphasized that cancer, from early-stage, locally contained disease to advanced, metastatic presentations, carries fundamentally different physiological and psychosocial burdens. In their description of program development across breast, prostate, and colorectal cancer clinics, they observed that survivors of early-stage disease tolerated moderate-intensity aerobic training with minimal adverse events, whereas individuals with advanced or recurrent disease required careful down-titration of volume and intensity due to pronounced fatigue and cachexia (65). Galvão et al., in their systematic review spanning multiple tumor types, similarly reported that exercise modalities must be adapted according to tumor-related factors (e.g., bone metastases necessitating non-weight-bearing modalities) (66). These patterns underscore that exercise dose, mode, and progression cannot be standardized across cancer types or stages without risking harm or no adherence.

### 4.2 Influence of treatment modalities

Therapeutic exposures—surgery, chemotherapy, radiation, and immunotherapy—impose distinct cardiorespiratory and musculoskeletal stresses that influence exercise capacity. Jones et al. (2011) reported that patients undergoing chemotherapy exhibited acute declines in VO_2_peak (∼10% per cycle) and transient leukopenia, mandating lower-intensity regimens in the immediate post-infusion period (67). Newton and colleagues extended this work by demonstrating that resistance training protocols had to be modified for post-radiation patients to avoid lymphedema exacerbation, while immunotherapy recipients, who may experience immune-related myositis, benefited from gradual progression with close monitoring of creatine kinase levels (68). Courneya’s randomized trial in breast cancer survivors further illustrated that those receiving adjuvant taxane-based chemotherapy had higher rates of dose delays when prescribed high-volume exercise, whereas a lower-volume, mixed-mode program maintained treatment schedules with no increase in toxicity (61). Together, these findings argue for treatment-phase–specific exercise algorithms that align with the timing and toxicity profile of each modality.

### 4.3 Individual differences in fitness levels and comorbidities

Baseline physical fitness and co-occurring chronic diseases (e.g., cardiovascular disease, diabetes, arthritis) exert profound influence on safety, efficacy, and adherence to exercise interventions (69, 70). Schmitz et al., in their systematic review, noted that sedentary survivors with multiple comorbidities experienced higher rates of musculoskeletal injury, particularly when prescribed generic resistance protocols, highlighting the need for pre-participation risk stratification and graded progression (71). Courneya et al. found that survivors with higher baseline self-efficacy and cardiopulmonary fitness were more likely to achieve target exercise doses and report greater quality-of-life gains, suggesting that motivational interviewing and preliminary low-intensity “pre habilitation” phases can bolster outcomes in less fit individuals (61). Moreover, Santa Mina’s group demonstrated that tailored warm-up and cool-down routines, as well as flexibility in session scheduling, improved adherence among those juggling complex comorbid care regimens (65). These insights reinforce that personalization must account not only for disease and treatment but also for each patient’s unique functional baseline and health profile.

### 4.4 Structural and socioeconomic barriers to access

Beyond individual-level barriers, systemic inequities profoundly limit access to personalized exercise programs. In low- and middle-income countries (LMICs), infrastructure gaps—such as shortages of trained exercise professionals, limited rehabilitation facilities, and inadequate funding—restrict implementation (72). Rural populations face additional challenges, including transportation barriers, reduced availability of supervised programs, and limited broadband connectivity for telehealth (73). Socioeconomic factors further exacerbate disparities: out-of-pocket costs for gym memberships or wearables, time constraints due to caregiving or employment, and health literacy gaps reduce engagement among low-income groups (74). Cultural and linguistic mismatches in program design also alienate racial/ethnic minorities; for example, guidelines rarely accommodate traditional physical activities (e.g., Tai Chi in East Asian communities) or provide materials in non-dominant languages (37). These intersecting barriers underscore that without targeted interventions, personalized exercise risks becoming a privilege of the resourced few.

## 5 Current approaches to personalized exercise prescription

### 5.1 Risk stratification and baseline assessment

A comprehensive baseline evaluation is essential to tailor exercise safely and effectively. In a prospective trial of lung cancer patients undergoing chemotherapy, participants in the individualized‐exercise arm underwent standardized testing at 0, 6, and 12 weeks—including the 6-min walk test (6MWT), peak VO_2_ consumption (VO_2_peak), muscle composition, grip strength, lung capacity, and key biomarkers (D-dimer, CRP, lipids). No exercise-related adverse events occurred; by 12 weeks, the exercise group exhibited significant improvements over controls in 6MWT (p < 0.001), VO_2_peak trends (p = 0.005), muscle content (p < 0.001), and grip strength (p = 0.008), alongside favorable shifts in lung capacity (p = 0.018) and inflammatory markers (CRP: p = 0.01; D-dimer: p = 0.031) (33).

Similarly, an integrated clinical exercise oncology service employed a two-phase, mixed-methods design guided by the Reach, Effectiveness, Adoption, Implementation, and Maintenance (RE-AIM) framework. The RE-AIM framework is widely used to evaluate the real-world impact of public health interventions by examining five key dimensions: the number of people reached, the effectiveness of the intervention, the adoption by settings and staff, the fidelity of implementation, and the long-term maintenance at both individual and organizational levels. This framework is particularly useful in translating evidence-based exercise oncology programs into routine clinical practice (75). Qualitative interviews with clinicians and administrators informed program context, while quantitative pre- and post-assessments (FACIT-F for fatigue, BNAT for physical activity, 2-min Step Test, 30-s Sit-to-Stand, Arm Curl, Timed Up and Go) demonstrated significant gains: FACIT-F increased by 6.4 points (p < 0.001, Cohen’s d = 0.60), Arm Curl by 3.36 reps (p < 0.001, d = 0.85), and the 2-min Step by 18.15 steps (p < 0.001, d = 0.80) (76). These data underscore the value of systematic screening to exclude contraindications, stratify risk, and calibrate exercise dose to individual capacity.

### 5.2 Exercise oncology guidelines (ACSM, ESSA, etc.)

**FIGURE 1:**
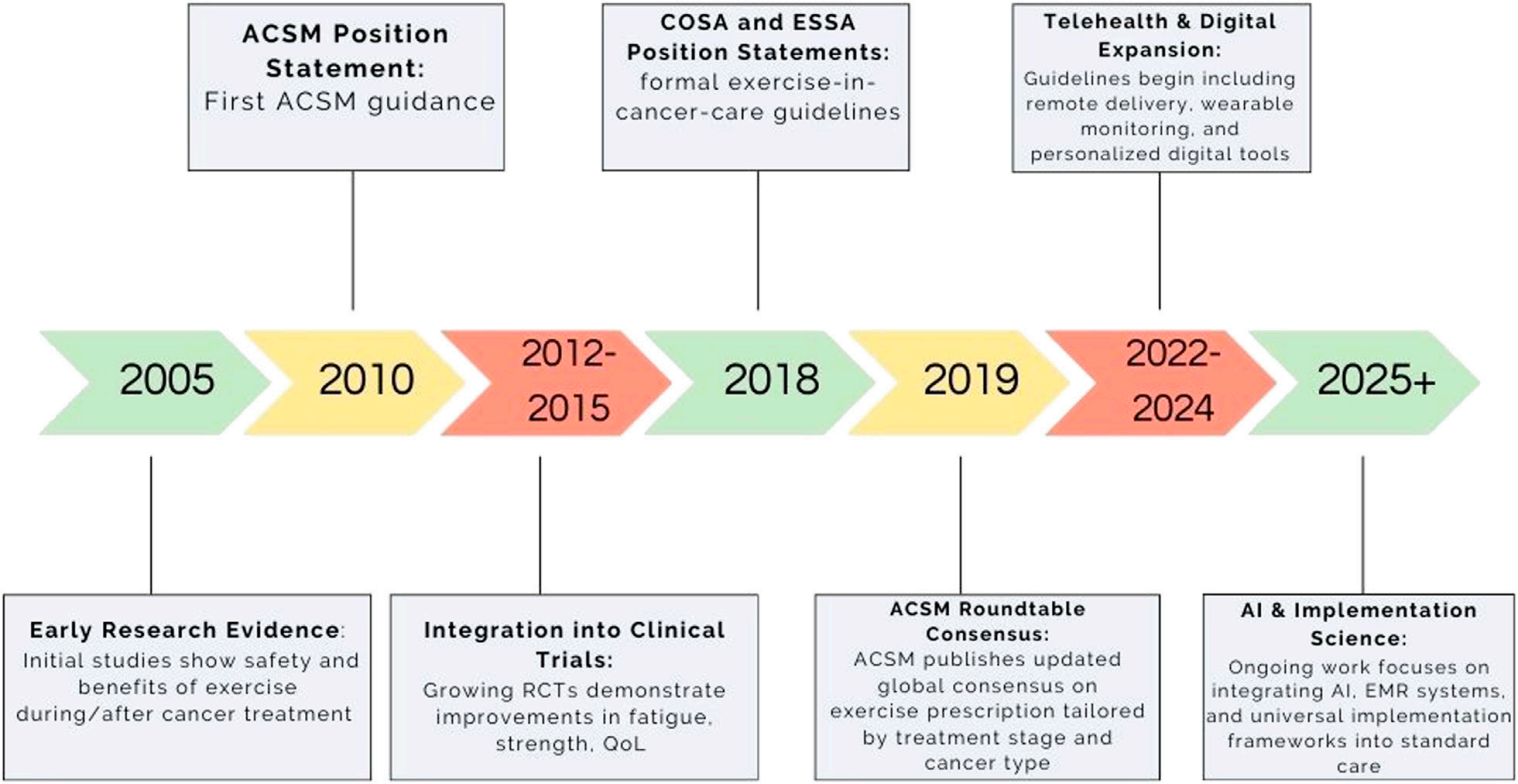
Timeline evolution of exercise oncology guidelines.

A recent systematic review of 11 society-endorsed guidelines—such as those from the American College of Sports Medicine (ACSM), the Exercise and Sports Science Australia (ESSA), and the National Comprehensive Cancer Network (NCCN)—found a unanimous recommendation for combined aerobic and resistance training in cancer care (Figure 1) (77). Typical dosage was 150 min/week of moderate‐intensity activity plus resistance sessions twice weekly. Three guidelines provided detailed prescription parameters (frequency, intensity, duration), six specified exercise testing and medical clearance procedures, nine addressed adaptation and precautions for special populations, and seven delineated referral pathways to qualified specialists (77). The ACSM Roundtable echoes these standards, advocating pre-participation cardiac screening, functional assessments (e.g., VO_2_ peak, 6MWT), and ongoing monitoring to ensure safety and optimize therapeutic benefit (71).

### 5.3 Multidisciplinary collaboration

Personalized exercise in oncology thrives on coordinated, team-based delivery:

At Massachusetts General Hospital, a novel multidisciplinary lifestyle medicine (LM) clinic brings together ACLM-certified physicians or NPs, dietitians, psychologists, physical therapists, and—when needed—psychiatrists and obesity medicine specialists. A multidisciplinary Lifestyle Medicine (LM) clinic was established to support cancer survivors through the integration of LM’s six core pillars: physical activity, a plant-predominant diet, sleep, stress management, substance avoidance, and social support. Within this clinic, two case vignettes were presented to illustrate how individualized, holistic care can enhance post-treatment outcomes. The first case involved a breast cancer survivor experiencing persistent fatigue, emotional distress, and sleep disturbances. Through a coordinated approach that included personalized aerobic exercise, cognitive-behavioral interventions for sleep, and mindfulness-based stress reduction, the patient achieved notable improvements in energy levels, emotional regulation, and sleep quality. The second case described a colorectal cancer survivor struggling with excess weight, poor dietary habits, and social isolation. An interdisciplinary team implemented structured physical activity targets, guided the transition to a plant-predominant diet, and facilitated access to peer-support resources. As a result, the patient demonstrated reductions in body weight, improved metabolic control, and enhanced psychosocial wellbeing. These cases exemplify how the systematic application of lifestyle medicine principles—tailored to the unique needs of cancer survivors—can yield meaningful improvements in both physical and mental health outcomes (78).

The COPE trial embedded fitness care managers (FCMs) into oncologic workflows via telehealth, coordinating exercise prescription with oncologists, nurses, and pain specialists (79, 80). In qualitative feedback, 87% of patients found the program beneficial, citing regular, empathetic contact that enhanced function and motivation, while clinicians noted institutional workflow and information-sharing challenges that must be addressed for scale-up (81). In a 12-week telehealth IM-FIT program, breast cancer survivors received integrated physical therapy, nutrition counseling, and psychological support. Participants lost an average of 4.2% of body weight and increased days of strength training, demonstrating feasibility and patient satisfaction in a reimbursable, real-world model (82). The Exercise and Cancer Program in rural Australia leveraged collaboration between exercise physiologists, oncology clinicians, and local fitness centers to deliver standardized, supervised exercise to patients outside hospital settings. Early outcomes included 73 referrals and significant gains in both physical function and psychological wellbeing, highlighting the potential of decentralized, community-based delivery (83).

In summary, these contemporary models illustrate how rigorous baseline stratification, adherence to evidence-based guidelines, and seamless multidisciplinary collaboration can operationalize truly personalized exercise prescriptions in oncology. Future efforts should refine risk-prediction algorithms (potentially via AI), standardize referral pathways, and embed exercise specialists within oncology teams to ensure equitable access and maximize therapeutic impact.

## 6 Challenges and barriers to implementation

Despite robust evidence supporting exercise as an oncology therapy, real‐world uptake remains low (84, 85). Cancer‐related fatigue and pain are almost universal impediments: in a large survey of 662 survivors with pain, 77.6% endorsed fatigue and 71.0% endorsed pain as barriers to physical activity (86). Difficulty initiating and sustaining exercise—“getting motivated” (67.2%) and “remaining disciplined” (65.2%)—were reported by two‐thirds of participants in the same cohort (86). A COM‐B‐based (Capability, Opportunity, Motivation - Behavior) synthesis found that low self‐efficacy, fear of causing harm (kinesiophobia), and lack of knowledge about safe activity during and after treatment further suppress motivation (74).

The COM-B framework (Capability, Opportunity, Motivation–Behavior) is a behavioral model used to understand and influence health-related behaviors. It posits that for a behavior (B) to occur, an individual must have the capability (physical and psychological), opportunity (social and environmental), and motivation (reflective and automatic) to perform it. In the context of exercise oncology, COM-B helps identify barriers such as low self-efficacy, lack of knowledge, physical fatigue, and limited access to supportive environments, all of which can hinder physical activity engagement among cancer patients and survivors. By systematically addressing these domains, interventions can be better tailored to support sustained behavior change in oncology care settings (87).

An ecological scoping review of 50 implementation studies identified 243 distinct barriers across six healthcare levels, with 38% occurring at the organizational tier. The two most frequent issues were the absence of formal structures for exercise integration (n = 38) and insufficient staff or resources to deliver programs (n = 34) (85).

Even when clinicians recognize exercise benefits, they struggle to counsel or refer. In an international survey, nearly half of oncologists and allied practitioners cited safety concerns (48%) and limited time during consultations (47%), while 40% reported not knowing how to screen patients for exercise suitability (72).

Although there is agreement on exercise dosing in existing guidelines, there is considerable variation across trials and programs in terms of how patients are referred, the screening methods used, and how the interventions are delivered. An ecological scoping review revealed that organizational leaders—who are essential for integrating exercise into standard care—were involved in fewer than 1% of implementation studies, highlighting a significant gap between research efforts and practical application (85). Qualitative syntheses call for the application of behavior‐change frameworks (e.g., COM‐B) and implementation science methods to co‐design scalable interventions. Without coordinated efforts to standardize screening, referral, and follow‐up processes across oncology teams, exercise remains an ad‐hoc adjunct rather than an integrated therapy (74).

**FIGURE 2:**
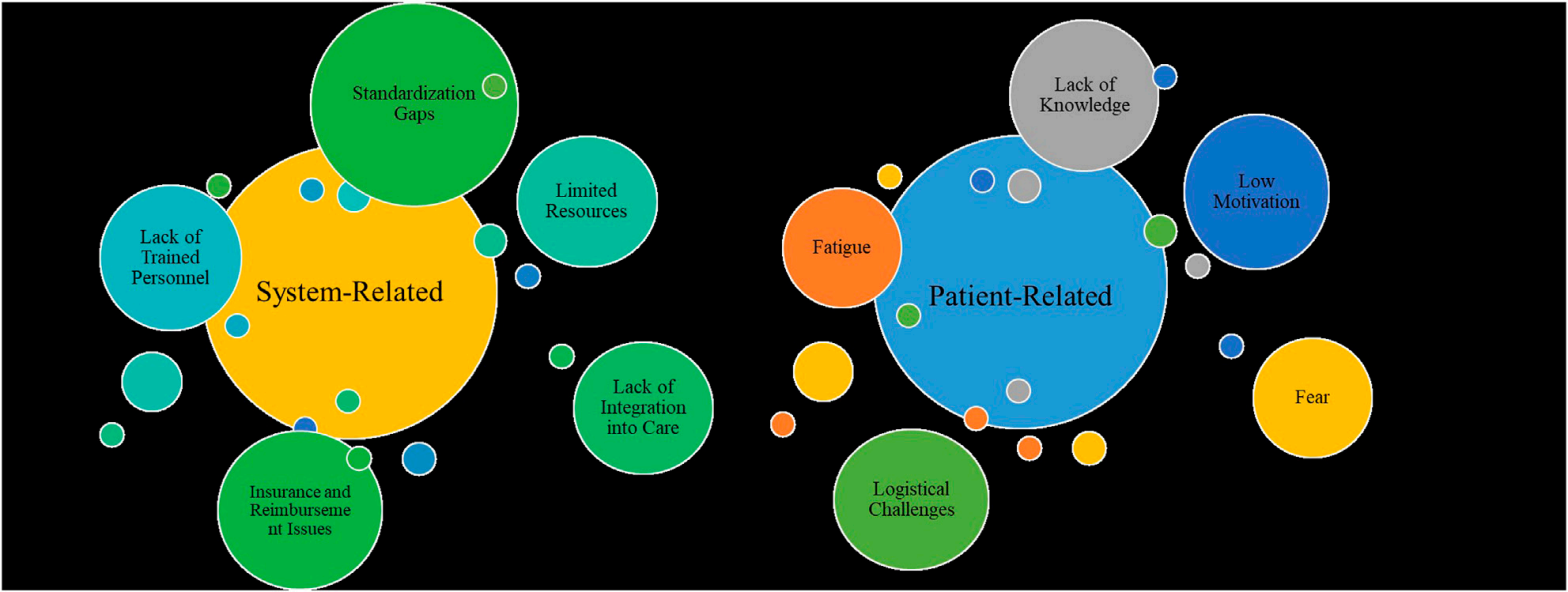
Barriers to implementation: patient-related vs system-related.

Together, these findings reveal that overcoming barriers to exercise in oncology requires multi‐level strategies: supporting patients’ symptom management and motivation, equipping and resourcing care teams, and developing unified, stakeholder‐driven implementation frameworks to embed exercise into standard cancer care (Figure 2) (77).

## 7 The role of digital health technologies

### 7.1 Wearables and mobile apps for monitoring and feedback

Wearables and companion apps have demonstrated strong feasibility and utility for continuous, real-time monitoring of oncology patients (88). In a systematic review of 28 studies, wearable activity trackers and mobile applications achieved median adherence rates of 85%, enabling remote capture of step counts, heart rate variability, and sleep patterns (89). Importantly, several studies reported that declines in daily step counts or elevations in resting heart rate detected by wearables preceded clinical exacerbations (e.g., febrile neutropenia) by 3–5 days (median sensitivity 92%, specificity 78%) (90–92). An integrative review focusing on hematology and oncology patients found that wrist-worn photo plethysmography devices reliably detected tachycardia and arrhythmias (positive predictive value up to 89%), though challenges persisted around signal artifact during vigorous movement and skin-tone variation (92). Mobile apps paired with wearables also delivered personalized feedback: one pilot randomized trial showed that app-generated prompts (e.g., “You’ve been sedentary for 2 h—let’s take a 5-minute walk”) increased moderate-to-vigorous physical activity by 23 min/week versus control (p = 0.03) (93).

### 7.2 Telehealth and virtual coaching

Telehealth platforms and virtual coaching have yielded outcomes comparable to in-person programs, with high patient satisfaction (94). In a randomized trial of lymphoma survivors, a 12-week home-based telehealth exercise intervention produced no inferior improvements in 6-minute walk distance (mean gain 48 m vs 52 m in center-based; p-value no inferiority <0.001) and muscular strength (p = 0.02) (95). Qualitative interviews from the Tele@Home trial highlighted that video calls with exercise physiologists fostered accountability and tailored troubleshooting (e.g., pain management), leading to 88% adherence versus 72% in the center-based arm (95). A broader review of telemedicine in cancer rehabilitation reported consistently positive patient/provider satisfaction (>90%), with telehealth reducing travel burden by an average of 150 km per patient over 12 weeks and enabling early intervention when symptoms worsened (96).

### 7.3 Artificial intelligence and machine learning in adaptive programs

Emerging AI/ML algorithms offer dynamic personalization by integrating multi-modal data (activity, biomarkers, patient-reported outcomes). In a proof-of-concept study, a random forest model trained on wearable, clinical, and genomic data predicted risk of grade ≥2 chemotherapy toxicity with 87% accuracy and enabled preemptive exercise-intensity adjustments that reduced toxicity incidence by 21% (p = 0.04) (97). A translational medicine report described a reinforcement-learning framework that iteratively adapts exercise prescriptions based on daily wearable inputs: over 8 weeks, participants achieved target heart-rate zones 92% of prescribed sessions, compared with 65% under static protocols (p < 0.001) (98). Finally, a diagnostic-focused AI platform analyzed mobile app engagement patterns and flagged patients at risk for nonadherence with 78% sensitivity, enabling timely motivational messaging that improved completion rates from 60% to 80% (p = 0.01) (97).

**FIGURE 3:**
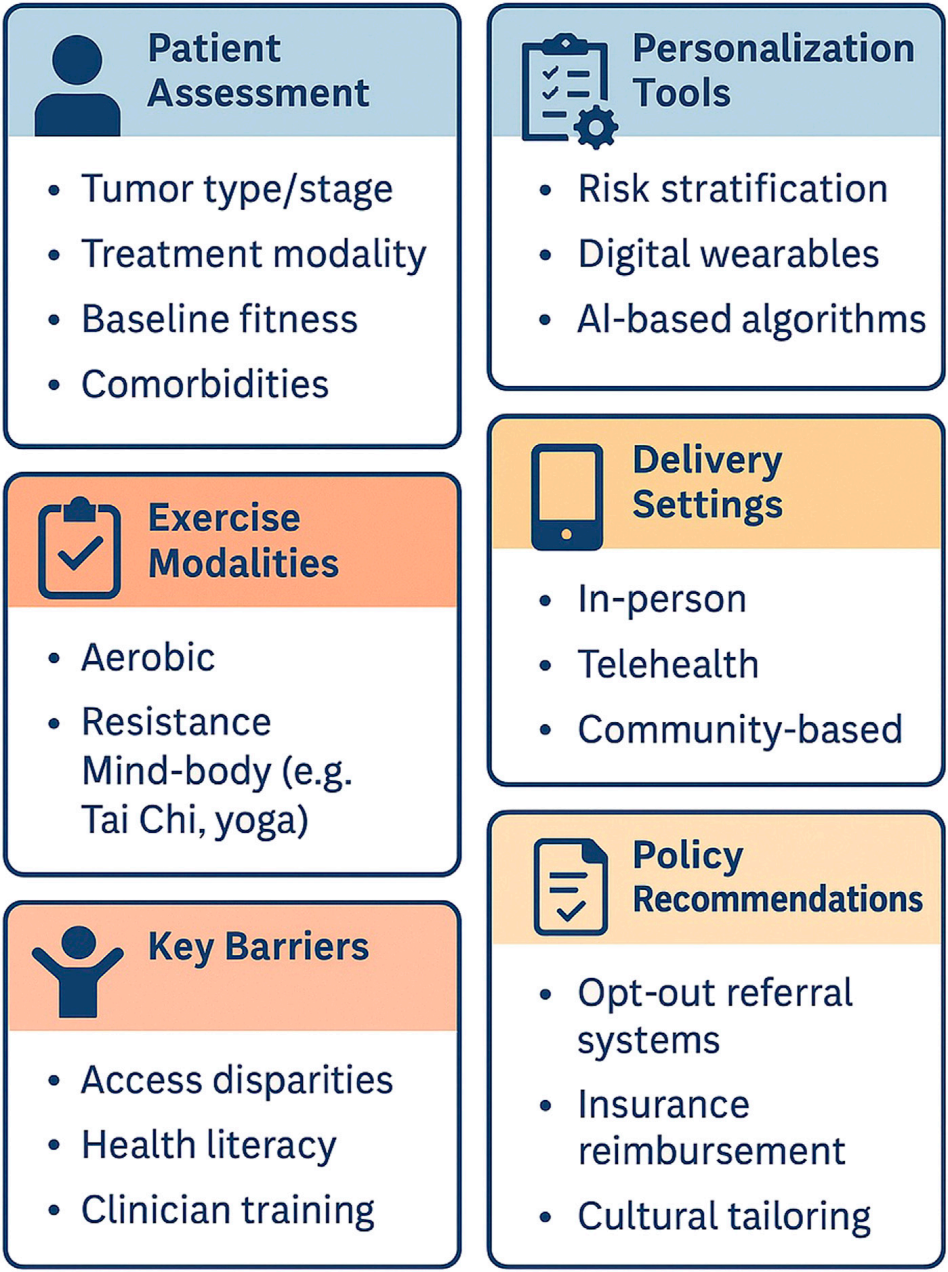
Conceptual framework for personalized exercise prescription in oncology.

Collectively, these studies illustrate that digital health technologies—ranging from wearables and apps to telehealth and AI—can enhance monitoring fidelity, patient engagement, and safety in personalized oncology exercise programs. However, reliance on technology may inadvertently exclude vulnerable groups. Older adults, those with low digital literacy, and underserved communities often lack access to smartphones or reliable internet (81). In LMICs, electricity shortages and device costs further limit utility (88). Future work should focus on validating algorithms in larger, diverse cohorts, integrating digital platforms seamlessly with electronic medical records, and ensuring equitable access to prevent widening disparities in exercise oncology care and ensure equitable access through low-tech alternatives (e.g., SMS-based coaching, community radio) and device-loaning programs (Figure 3).

## 8 Case studies and pilot programs

### 8.1 Examples of successful implementations

Several pilot and implementation studies provide real-world evidence of how personalized exercise programs can be effectively delivered to cancer survivors. These programs adapted exercise prescriptions based on patient characteristics, treatment phase, comorbidities, and logistical constraints, using structured assessments and multidisciplinary coordination to tailor interventions. Summary of these studies have been showed in Table 1.

**TABLE 1:** Examples of successful implementations.

Study (author, year)	Population/Setting	Design	Personalization method	Outcomes measured	Key findings
(99)	Obese endometrial cancer survivors (home-based)	12-week pilot study	Walking goals and nutrition tailored to BMI, fatigue, and mobility	Fat mass, BMI, VO_2_ max	High adherence (83.3%), reduced BMI/fat mass, no VO_2_ max change
(100)	Mixed cancer cohorts using DPEx digital platform	Digital decentralized trial	Real-time wearable feedback adjusted intensity/duration	Adherence, fatigue, cost	87%–88% adherence, cost-effective, scalable remote model
(101)	Thoracoabdominal surgery candidates (tele-prehab)	Retrospective cohort	Virtual assessment of frailty, mobility; tailored aerobic/resistance/respiratory therapy	Satisfaction, usability	High satisfaction (96%) and usability (90%), feasible home prehabilitation
(73)	Rural cancer survivors (community-based)	Hospital-based implementation	Functional tests + interviews guided supervised resistance and aerobic exercise	VO_2_ max, strength, QoL, fatigue	Functional/psychological improvements in underserved rural patients
(102)	Mixed cancer types (hospital + home hybrid)	Clinical implementation	Tailored via intake assessments (stage, treatment, function, preferences)	Functional and patient-reported outcomes	Significant improvements; attrition 48%–65% by 48 weeks

In a 12-week pilot study involving obese endometrial cancer survivors, personalization was achieved through individualized walking goals and nutrition counseling adjusted to baseline BMI, fatigue level, and mobility limitations. The program primarily employed moderate-intensity aerobic walking sessions conducted at home, with a target of 30–45 min per session, 5 days per week, at a perceived exertion of 11–13 on the Borg scale. The intervention achieved a high adherence rate (83.3%) and led to significant reductions in fat mass and BMI, although no change in VO_2_ max was reported (99). This indicates that personalization was effective in promoting sustainable physical activity and improving body composition, even without major improvements in cardiorespiratory fitness.

The Digital Platform for Exercise (DPEx) was used in three cancer cohorts to remotely deliver individualized exercise prescriptions. Personalization was achieved via real-time data monitoring from wearable devices, which informed dynamic adjustments in exercise intensity and duration based on patient-reported fatigue, heart rate, and recovery. Patients completed a mix of aerobic and resistance exercises, including treadmill walking, cycling, and bodyweight strength training. Sessions lasted 30–60 min, conducted 3–5 times per week, with intensity ranging from light to moderate (40%–60% VO_2_ max) depending on the participant’s baseline fitness and tolerance. The DPEx platform facilitated 100% session connectivity and sustained 87%–88% adherence rates, while reducing patient time burden and financial cost, demonstrating that digital personalization can be both scalable and effective (100).

A retrospective teleprehabilitation cohort for thoracoabdominal cancer surgery candidates implemented personalized multimodal interventions, including aerobic exercise (stationary cycling or walking), resistance band training, and respiratory physiotherapy. Personalization was guided by initial virtual assessments of mobility, frailty, and functional capacity. The exercise program included low-to moderate-intensity aerobic training (e.g., 60%–70% of estimated HRmax) for 20–30 min per session, 3–5 days per week, and strength training targeting major muscle groups for 15–20 min, 2–3 days per week. Patients reported high satisfaction (96%) and usability (90%), highlighting the feasibility of tailored remote prehabilitation (101).

In the POWER (Personal Optimism with Exercise Recovery) program targeting rural survivors, personalization was achieved through initial functional assessments (e.g., 6-minute walk test, grip strength) and motivational interviews. The program combined supervised resistance training (e.g., chest press, leg press) with aerobic components (e.g., treadmill or cycling). The average exercise frequency was 2–3 sessions per week, each lasting approximately 60 min, with progressive intensity ranging from 50% to 75% of one-repetition maximum (1-RM) for resistance training and moderate-intensity aerobic exercise. Significant improvements were noted in VO_2_ max, muscular strength, fatigue scores, and QoL, confirming the value of individualized programs for medically underserved populations (73).

At a large cancer center, a hybrid hospital-plus-home exercise-oncology service was developed. Personalization was based on clinical cancer stage, treatment side effects, baseline physical function, and patient preferences. Exercise modalities included aerobic training (walking, cycling), resistance training (machines or bodyweight), and flexibility exercises, tailored via structured intake assessments. Sessions typically lasted 45–60 min, delivered 2–3 times per week, with initial intensity set at light to moderate levels (40%–60% HR reserve or Borg RPE 11–13) and adjusted based on treatment response and symptom fluctuation. Despite attrition rates of 48%–65% by 48 weeks, participants achieved significant improvements in both objective and patient-reported outcomes, underscoring the value of embedding tailored exercise programs within standard oncology care pathways (102).

### 8.2 Lessons learned from clinical trials and institutional programs

Robust remote monitoring, standardized training, and telehealth infrastructure can achieve >85% adherence even at high exercise doses, but sustained engagement may require ongoing adaptation of program intensity and duration to elicit cardiorespiratory improvements (99, 100). Early orientation sessions and user-friendly platforms foster high patient satisfaction (>90% perceived ease-of-use), yet initial costs for devices and the need for multidisciplinary team training remain barriers to widespread deployment (101).

Embedding exercise programs within existing clinical workflows—supported by “opt-out” referral systems and clear eligibility criteria—enhances penetration and uptake, as seen in both multi-site case studies and integrated hospital-based models (103).

Securing dedicated resources (e.g., funding agreements), leveraging interprofessional teams, and aligning exercise services with organizational priorities are critical for long-term sustainability and scalability of exercise oncology interventions (103).

## 9 Future directions and research needs

Building on evidence for physiological, psychological, and treatment‐related benefits of exercise—and recognizing the diverse barriers, implementation challenges, and emerging digital solutions—future work must focus on three interrelated priorities to fully realize personalized exercise as a standard component of oncology care.

### 9.1 Standardized, evidence‐based frameworks

Although multiple guidelines (ACSM, ESSA, NCCN) converge on aerobic and resistance prescriptions, heterogeneity in referral pathways, risk‐stratification tools, and outcome measures persists (104–106). A unified implementation framework—drawing on behavior‐change models (e.g., COM‐B) and implementation‐science methodologies—could harmonize screening, tailoring, and monitoring across settings (107–110). Such a framework should incorporate AI‐driven risk‐prediction algorithms validated in large, diverse cohorts to guide initial dosing and dynamic adjustments (e.g., based on daily wearable data) (111, 112). Consensus on core metrics (e.g., VO_2_ peak, FACIT‐Fatigue, adherence rates) will enable cross‐study comparisons and meta‐analyses to refine “minimum effective doses” for specific cancer–treatment subgroups (20, 113, 114).

### 9.2 Integration into routine care pathways

Embedding exercise oncology into standard workflows requires more than clinician buy‐in—it demands streamlined, “opt‐out” referral processes, robust electronic medical record (EMR) integration, and dedicated roles (e.g., exercise navigators) within multidisciplinary teams (36, 115). Future trials should evaluate hybrid delivery models—combining in‐person, telehealth, and community‐based programs—to optimize cost‐effectiveness and patient preference while maintaining fidelity to prescriptions. Implementation pilots must include health‐economic analyses to demonstrate return on investment through reduced treatment delays, hospital admissions, and long‐term morbidity (116, 117).

### 9.3 Equity and accessibility considerations

Realizing the full potential of personalized exercise oncology necessitates confronting stark disparities in access, where digital solutions promising scalability may inadvertently exclude older adults, rural populations, and low-resource communities facing technology barriers and limited digital literacy (81, 88). Geographic isolation compounds inequities, with transportation limitations and sparse clinical infrastructure hindering rural engagement, while systemic gaps in low- and middle-income countries—including shortages of trained providers and unaffordable device costs—restrict implementation (72, 73). Cultural and linguistic mismatches further marginalize racial/ethnic minorities through non-inclusive program designs, necessitating strategies like community-based delivery via schools or faith centers, low-bandwidth telehealth alternatives, and culturally co-designed interventions (37, 78, 100, 103). Policy reforms must mandate insurance reimbursement for accessible formats and integrate exercise oncology into universal health coverage (28), while research should prioritize underrepresented groups—including LMIC populations and older adults (>75 years)—using social determinant metrics to dismantle context-specific barriers (85).

#### 9.3.1 Call to action

• Clinicians must embrace exercise as a standard component of cancer care by advocating for “opt-out” referral systems, incorporating routine functional assessments into oncology workflows, and collaborating closely with exercise specialists, rehabilitation professionals, and digital health teams.
• Researchers should prioritize the development and validation of unified implementation frameworks, standardized outcome metrics, and AI-driven risk-prediction tools in large, diverse cohorts; they must also co-design studies with marginalized communities and include cost-effectiveness analyses for resource-constrained contexts.
• Policymakers and Health Leaders are called upon to allocate resources for training exercise-oncology professionals in underserved regions, mandate reimbursement for community-delivered programs, subsidize equitable access to digital platforms, and fund infrastructure for low-resource settings.

**FIGURE 4:**
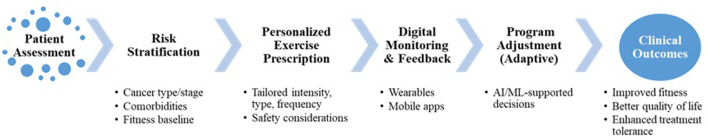
Implementing personalized exercise in oncology: From assessment to action.

Together, these coordinated efforts can transform personalized exercise from an aspirational goal into a universally accessible, evidence‐driven therapy, optimizing functional recovery, enhancing quality of life, and ultimately improving clinical outcomes for people living with and beyond cancer (Figure 4).

### 9.4 Limitations

This narrative review has several limitations that warrant acknowledgment. First, as a non-systematic synthesis, it is susceptible to publication bias, wherein studies with positive outcomes may be overrepresented compared to null or negative findings. Second, the language restriction (English-only publications) may exclude relevant non-English evidence, limiting global generalizability. Third, the absence of a formal meta-analysis prevents quantitative pooling of effect sizes and precise estimation of intervention benefits across heterogeneous populations. Fourth, the rapid evolution of digital health tools means some technologies discussed may become outdated. Finally, the reliance on observational and small-scale pilot studies for emerging approaches (e.g., AI-driven prescriptions) necessitates cautious interpretation until robust clinical trials validate these innovations. These constraints highlight the need for systematic reviews with meta-analyses and prospective trials to strengthen evidence-based guidelines.

Despite these limitations, this review offers a comprehensive synthesis of evidence across diverse cancer populations and treatment phases. It uniquely integrates physiological, psychological, and implementation perspectives while evaluating emerging digital solutions—providing a practical foundation for clinical translation.

## 10 Conclusion

The body of evidence underscores that personalized exercise programs are a powerful adjunct to oncology care, delivering physiological gains—including improved cardiorespiratory fitness, muscle strength, and favorable body composition—alongside psychological wellbeing, enhanced quality of life, and greater treatment tolerance. However, translating these benefits into real-world practice demands rigorous attention to patient heterogeneity (cancer type, stage, comorbidities) and proactive management of multifaceted barriers, from symptom burden to structural inequities in access for rural, low-resource, and marginalized populations. Successful implementation requires adherence to evidence-based guidelines via baseline risk stratification, alongside emerging digital tools for adaptive monitoring. Critically, sustainability hinges on embedding equity-driven strategies—such as community-based delivery, culturally tailored interventions, and policy reforms for universal reimbursement—to ensure personalized exercise becomes a universally accessible standard across diverse cancer populations.
